# COVID-19 research response to immediate demands: setting priorities with key stakeholders to enable health services research in NSW, Australia

**DOI:** 10.1108/JHOM-03-2023-0059

**Published:** 2024-09-17

**Authors:** Nicole M. Rankin, Don Nutbeam, Jean-Frederic Levesque, Henry Ko, Garry Jennings, Adam Walczak, Christine Jorm

**Affiliations:** Evaluation and Implementation Science Unit, Centre for Health Policy, The University of Melbourne, Parkville, Australia; Sydney School of Public Health, The University of Sydney, Sydney, Australia; Agency for Clinical Innovation, New South Wales Ministry of Health, North Sydney, Australia; John Hunter Health and Innovation Precinct, The University of Newcastle, Newcastle, Australia

**Keywords:** Quantitative techniques, Health services, User participation, Priorities

## Abstract

**Purpose:**

COVID-19 has caused unprecedented disruption to health systems. There is much to be gained by capturing what was learned from changes and adaptations made by health services and systems. The Ministry of Health in New South Wales (NSW), Australia, sought to prioritise health services research (HSR) to address critical issues arising from the COVID-19 pandemic. We tested a priority setting methodology to create priorities for a specific funding opportunity and to extract generalisable lessons.

**Design/methodology/approach:**

A virtual roundtable meeting of key stakeholders was held in June 2020. We used a modified Nominal Group Technique (NGT) for priority setting, with potential items (*n* = 35) grouped under headings. Data was analysed through a reflective deliberative process.

**Findings:**

We engaged 89 senior policy makers, health service executives, clinicians and researchers in the roundtable. The NGT proved an efficient method with participants reaching consensus on eight priorities. Findings included strong support for learning from the rapid response to COVID-19 and addressing needs of vulnerable populations and the health workforce. Opinions differed about strategic areas investment and where learnings should be via internal evaluation rather than funded research. Three of the eight recommended priorities were included in the funding opportunity.

**Research limitations/implications:**

Coronavirus disease 2019 (COVID-19) required unprecedented change and adaptations within health systems, and rapid, applied health services research can help to create, understand and (where relevant) sustain change beyond the immediate impact of the pandemic. While final decisions may be dependent on a wider range of considerations by government, stakeholder enthusiasm for engagement in priority setting exercises may be dampened if they do not perceive their application in decision-making.

**Practical implications:**

A modified nominal group technique can be used to set research priorities in constrained conditions by engaging large numbers of stakeholders in rankings and then using an online delivery of a roundtable and to reach consensus on priorities in real time. Recommended priorities for health services research can be readily generated through rapid engagement but does not guarantee their application.

**Social implications:**

Australia’s swift response to COVID-19 pandemic in 2020 was perceived as a relative success due to the rapid public health and policy response and a relatively low number of cases. This response was underpinned by systematic knowledge mobilisation including support for targeted and prioritised health services research to fill knowledge gaps.

**Originality/value:**

Setting priority processes can provide rich, engaged input to support government funding decisions about HSR. A wide range of dynamic and iterative processes influence decision-making in a rapidly evolving situation in the health system response to COVID-19. It is crucial to consider how major investment decisions will support a value-based healthcare system.

## Introduction

Since early 2020 the COVID-19 pandemic has caused unprecedented health, economic and social upheaval across the world. The disruption has prompted change and adaptation in almost all human activity, including change at an unprecedented pace and scale in health services and systems. Some countries have seen their health systems overwhelmed by the pandemic. Australia, however, experienced the first wave of the pandemic in 2020 quite differently to many other countries, with time for testing and adapting preparedness measures due to the early closure of borders and tight controls over quarantine processes preventing high numbers of COVID-19 cases.

In the Australian response to the pandemic, new models of care were introduced across health services from early 2020, the most prominent being a reduction in face-to-face consultations through wider use of telehealth, remote monitoring of health conditions and the rapid scale-up of “hospital in the home” services (Monaghesh and Hajizadeh, 2020; Dickson, 2020; Montalto *et al.*, 2020). However, elective surgery was postponed, preventative healthcare programs suspended or reduced, and changes in patient behaviours included a reduction in hospital presentations for emergency care (Soltany *et al.*, 2020; Søreide *et al.*, 2020; Dragovic *et al.*, 2020). Clinical and health services research was significantly disrupted dislocation of activity with, for example, the cessation or significant disruption of clinical trials observable across many countries including Australia (Sutherland *et al.*, 2020; Levesque and Harris, 2020; Asaad *et al.*, 2020).

In New South Wales (NSW), Australia’s most populated state (8.09 million people), the initial response to the pandemic has greatly strengthened the link between health science and its rapid application to health policy and practice, with positive collaboration across health sectors (government, academia and clinical services) and engagement of agencies (Hyland-Wood *et al.*, 2021). This sits in contrast to a past often characterized by poor alignment of research activity with health services and public policy needs, made worse by limited mechanisms to review, disseminate and implement research within the health system (Jorm *et al.*, 2008; NSW Health, 2012; Department of Health and Ageing, 2013).

There are long-term benefits to be gained by recording, reviewing and learning from the forced and rapid adaptation to health services and systems that occurred. During 2020, the NSW Health Ministry (hereafter NSW Health) rapidly adopted entirely new ways of working in response to the pandemic, building on progressive reforms to improve the quality and use of information to support a “learning health system” (NSW Health, 2017). This included the formation of a new organizational entity – the Critical Intelligence Unit (CIU) – which brought together clinical, analytic, research, organizational and policy experts to provide timely and considered advice in response to the pandemic (Levesque *et al.*, 2020). The CIU’s work was based on the principal of “satisficing” in order to provide “good enough” advice in a situation where evidence was limited and changing rapidly (Levesque *et al.*, 2020). Our team sought to bring together senior leaders from multiple NSW-based agencies, inclusive of clinical health services, policy makers and the research community, to review and rapidly define priorities for COVID-19 related rapid applied research. Research priority-setting is a growing field of endeavour (Yoshida, 2016) that seeks consensus about areas where increased research effort (including collaboration, coordination and investment) will have wide benefits (Bryant *et al.*, 2014). There is a lack of knowledge about which methodology to use under different conditions and commonly used techniques have recognised strengths and limitations (Bryant *et al.*, 2014). Priority-setting processes are typically time consuming for participants, whether face to face (Synnot *et al.*, 2019), on-line (Smith *et al.*, 2020) or that use a combination (Richardson *et al.*, 2020). The James Lind Priority Setting Partnership Process typically takes 12–18 months (Nygaard *et al.*, 2019).

The COVID-19 pandemic created a necessity to invest in health services research that would help foster an understanding of the changes in health systems that worked well, delivered safe, quality health care and could be continued or expanded, as well as those which should not continue or where further development is needed, or where unintended consequences need to be addressed (Moynihan *et al.*, 2021). Lessons learned from responses to COVID-19 could contribute to an understanding how to create a more resilient health system (Clay-Williams *et al.*, 2020).

We designed a priority setting roundtable exercise to guide decision making by NSW Health and provide recommendations about investments in future health services research (HSR) funding. A further objective was to test the priority setting methodology and to extract any generalisable lessons that could inform strategic priority setting for HSR. This paper describes the priority setting process, the recommendations formulated and subsequent decisions taken to fund projects, and articulate what lessons were learned about that might be useful and generalisable for future HSR priority setting exercises.

## Methods

### Context and scope

This work was conducted in NSW, Australia, under the auspices of NSW Health. Australia is a federation, and the Commonwealth Government is responsible for primary care and the States for tertiary and community healthcare (Levesque *et al.*, 2020). Organisation of the roundtable commenced in May 2020 and was completed in mid-July 2020, at which time about 1000 COVID cases and 109 deaths had been experienced in NSW (Andrikopoulos and Johnson, 2020). Across this period, there was an intense sense of anxiety in the wider community with uncertainty about the spread of COVID-19, projected modelling of case numbers, and healthcare workers were experiencing significant psychosocial distress (Smallwood *et al.*, 2021). Policymakers and CIU experts were highly cognisant of the important roles of knowledge, evidence and learning in this period and the imperative to care for staff was balanced alongside testing new models of care (Levesque *et al.*, 2020).

The scope of this work was to determine research priorities for a second round of COVID-19 grants offered by NSW Health, which were designed to (1) support research into medium and longer-term issues related to the COVID pandemic in patients, the community and the health system; and (2) reduce the time from evidence generation to implementation (with a focus on the rapid planning, conduct and reporting of research, so that significant findings could be rapidly translated into clinical practice and policy) (NSW Health, 2020). These grants were intended as a mechanism to build capacity and collaboration in HSR across NSW and to ultimately benefit the population of NSW.

### Participants

Participants were healthcare stakeholders with a range of expertise including health service executives and managers at various levels within NSW Health, across Local Health Districts and frontline services, as well as clinicians and researchers with expertise in health services research.

Two strategies were used to identify participants. First, the NSW’s accredited Research Translation Centres (Robinson *et al.*, 2020): NSW Regional Health Partners; Maridulu Budyari Gumal (SPHERE); and, Sydney Health Partners, together with the centre “Health ANSWERS”, provided leadership in identifying researchers, clinicians and program/policy managers from their partner organisations (Local Health Districts, Universities and Medical Research Institutes). Second, leaders from the CIU and the NSW Health Office of Health and Medical Research (OHMR) nominated key executives. Key stakeholders were asked to nominate potential participants from the executive members of relevant public health organisations (e.g. Health Services Research Association of Australia and New Zealand). A select, senior and balanced group were personally invited. No reimbursement was offered for participation.

The authors adapted a methodologically robust approach for priority setting, establishing a collaborative group who were committed to ensuring that improved health system resilience should be a legacy of the COVID-19 challenge. We drew on existing expertise and considered some of the contemporary methodological challenges in priority-setting, including that such activities are frequently time consuming for participants (Bryant *et al.*, 2014). A modified nominal group technique (NGT) was the selected method, based on its merits of giving an equal voice to all participants and being sufficiently flexible for rating a large number of priorities within a single roundtable (Rankin *et al.*, 2016).

The NGT is a formal method for reaching consensus that was originally developed in the 1960s (Harvey and Holmes, 2012). It has been modified to suit broad areas of health and medicine, education and justice research. Four core components of the technique are defined as: problem identification, generation of appropriate research questions; opportunity to develop potential solutions; and reaching consensus on priorities for action (Harvey and Holmes, 2012). The selection and defining of criteria to assess solutions enable group discussion to ensure that important considerations about the context are not overlooked; examples include benefit (e.g. *will this benefit our community*?) and feasibility (e.g. *can we achieve the desired change*?) (Viergever *et al*., 2010). Modified NGT has been conducted as a single or series of face-to-face workshops. Since the COVID-19 pandemic, online consensus workshops have become more common (Hall *et al*., 2021; Mason *et al*., 2021). Given lockdown restrictions, the team had to engage participants in an online Zoom session, delivering a roundtable under significant time constraints. We had not previously attempted to apply the modified NGT methods in an online setting.

### Identification and collection of research priorities: initial collection of priorities

The collection and refinement of items were undertaken by two authors by initially considering 120 items included by the US Academy Health in a similar priority setting activity (DeCosta *et al.*, 2020). This list had been widely circulated in NSW. However, on detailed examination, few items were found to be relevant to the Australian context because of significantly different structural and funding models. This list was supplemented by research questions from the CIU and a total of 155 items were reviewed. Through iterative rounds of review to remove duplicate and redundant items (by all authors), a final list of 35 items were agreed. These were grouped under seven headings from the COVID-19 System Shock Framework (Hodgins *et al.*, 2021) (adapted from Hanefeld *et al.,* 2018): (1) health system response to the pandemic; (2) patient and community experience including mental health; (3) medical products, technologies and information systems (digital health); (4) health workforce; (5) health policy, governance and whole-of-government response; (6) funding and finance; and (7) health system values including an equity lens.

### Development of rating criteria

Two authors drafted rating criteria, which focused on significance to HSR and suitability for the grant round. The “significance” criteria were defined as “research that would contribute to *creating a resilient NSW health system, building a research legacy for NSW, generating research that fosters a* *‘learning health system’* *or contributes to values-based care*”. The “Round 2” criteria were defined as “research that would be considered as *feasible, supported by available data, good value for money, a research strength (for yourself, within LHD, or within NSW), and directly translatable into supporting the COVID-19 response in NSW*”. The rating criteria was reviewed by four authors before sending out a pre-roundtable survey.

### Pre-roundtable survey

Participants were invited to individually rate all 35 items via a survey two days prior to the roundtable. An email link to an online survey built in Microsoft Forms was circulated using the criteria described above.

### Roundtable activities

The authors developed a roundtable program and a set of resources to assist breakout group chairpersons and secretaries. Roles and responsibilities for the convenor and the group leaders were defined and shared. A final list of participants was reviewed and allocated into seven groups (11–12 participants in each) to ensure a mix of professions, gender representation and expertise. The authors consider that this pre-allocation would have positively impacted on the process, as participants were not grouped with their own work colleagues, giving participants an opportunity to openly share their views and opinions. As all 35 items needed to be rated within a 1.5-h roundtable, groups were allocated one of the seven themes and asked to rate between 4 and 8 items (score each item from 1 to 7, where 7 was highest priority) and then rank order the seven items in order of importance (from 1 most important to 8 least important).

### Break-out groups

The facilitated discussion in the break-out groups included sharing the pre-survey ratings. The priority setting tasks were described and time was allocated to enable participants to discuss and add new items. The process devised allowed for thoughtful and prepared discussion and each group had time for debate and reflection. Break-out groups concluded after 45 min, and participants reconvened in the main session to hear feedback from the group chairpersons. There was no completion of any conflict of interest statements prior to the workshop. It was anticipated that participants would be in the role of representing their employer organisation and would reflect on research priorities from this perspective. There was no process outline for the resolution of disagreements during the discussion.

### Data sources and analysis

*Roundtable:* An audio recording of the roundtable was transcribed verbatim. Two authors collated all quantitative rating data and qualitative analysis of contributions. A consultation report was produced by one author within four days of the roundtable and was circulated to all participants, providing a final opportunity to reflect on the priorities and provide feedback. A final report was prepared and submitted to NSW Health within three weeks of the roundtable.

The REPRISE reporting guidelines for research priority setting exercises (Tong *et al.*, 2019) have been used to structure this manuscript and a checklist version demonstrating completeness was completed.

## Results

### Participants

Table 1 shows a breakdown of professional groupings for the 89 participants, who were from clinical, academic, health policy and administration backgrounds. Participants demographics show seniority of positions (chief executive officers [CEOs], deputy CEOs or directors) to indicate engagement across health services. Fifty-five per cent of participants were female. An overview of the priority setting process from inception through to lessons learned is shown in Table 2. A thorough description of the selection, refinement and prioritisation of items across the project as well as a full list of all ranked items are included as Tables III and IV as a Supplementary File.

### Recommended priorities

A summary list of recommended priorities is shown below. A final list of items recommended as research funding priorities was collated, included in a draft consultation report and sent out to all participants after the roundtable. The top-rated items were:

(1) *Health system response:* Was the “cascaded planning approach” ([national], state, LHD, hospital plans) the best way to plan a response to the pandemic? Did it work equally well at state, LHD and hospital levels, and for urban, regional, rural and remote services? What variations occurred? Were some planning and implementation approaches more effective than others?
(2) *Health services response:* Conduct rapid cycle evaluations of major affected clinical services. How have they been impacted by COVID-19? Are there changes that should be sustained? Assess health economic impact of reconfigured services. Are there remedial actions we need to take to protect patients who missed out on care?
(3) *Health services response:* How do we make sense of the reduced visits to emergency departments and GPs during the COVID-19 scare? (Are there strategies that can help more patients manage outside the hospital system in “normal times? Understand what strategies the patient employed rather than attending ED and determine if the patient experienced a deterioration in their health and wellbeing.)
(4) *Mental health service response:* What were the determinants of successful interventions to support the mental health needs of vulnerable populations during the COVID response? What was the impact of location on interventions for mental health support for particular population groups, including regional and rural communities?
(5) *Medical technologies:* Which digital modality should we be using for which purpose/cohort? How can we optimise patient and clinician experience using digital modalities?
(6) *Health services response - priority populations:* How were the needs of priority populations within wider state-wide COVID-19 planning addressed? What methodology is in place for determining priority populations and did they prove to be the right priorities for the situation? And how would we translate such criteria to any future pandemics or other environmental challenges (like the bushfires)?
(7) *Workforce:* Evaluate the extended scope of practice developed in response to COVID-19 and consider how this could be supported/facilitated to provide surge capacity for future crises including study of the mental health impact on staff.
(8) *Primary Care:* How can primary care, the community health sector and residential aged care facilities, non-health government agencies and NGOs be integrated more expediently with emergency and acute health services to create a whole-of-civil-society response when needed?

### Qualitative findings

While there was much agreement about priorities within the groups, there were some aspects of setting priorities for this research funding opportunity that proved to be challenging. The concerns fell into four categories:

(1) Not a strategic investment

Some participants did not consider particular areas of research as being a priority for this funding opportunity. This included areas such as diagnostics and public health messaging, as proposals in these had already been sought in the first round COVID funding opportunity. Two further arguments were made during the discussion, first that there are other potential funders for some proposed research, for example, three participants said:

The Commonwealth [government] will evaluate the telehealth [reimbursement] items’ (Group participant) or “I would delete all items that could be funded from other sources … mental health will have access to alternative funding via the Commonwealth” (pre-roundtable survey response).

Secondly it was suggested that some topics were too large in scope for the NSW Health funding available. For instance, when the possibility of researching improvements to clinical data to develop real time learning analytics was discussed, this was considered just “*too hard*”.

(2) Tensions of terminologies: internal “evaluation” and “research”

Across the groups’ dialogue, there were differences in interpretation and meanings ascribed to “evaluation” and “research”. With regards to evaluation and research, there was feedback within several groups that items which would already be evaluated by NSW Health were of lower priority, but in other areas research was needed and should be prioritised. For example, one participant commented:

Research is not needed because we are doing an evaluation on that (within NSW Health)’ (Participant Group 3); this view was reinforced in written comments: “some of the policy priorities – this should be a natural quality improvement process” (pre-roundtable survey response).

In one group, a strong difference in views emerged, with some participants expressing that the early impacts of COVID-19 required further investigation from within services:

operational and systems-level analysis within the health system rather than research – and this analysis should be done by the health services.

As the discussion unfolded, this tension continued when an item about reductions to emergency admissions was discussed. The group rated this item as most important but again there was disagreement about the place of evaluation versus research. For example, one participant said:

Existing health services data systems and reporting cant answer these types of questions; (we) need new data through focused research (such as patient surveys).

However, group members were united in their views that not only looking retrospectively at data but also planning prospectively to examine changes in health service usage would be of vital importance for future pandemics. This included understanding why patients were making decisions about accessing services, changes in behaviour and the consequences on their health.

Within other groups, suggestions for internal evaluation purposes included longitudinal datasets to evaluate direct and indirect COVID impacts. In the case of virtual care it was noted that internal evaluation was ongoing, but one group participant thought it was important to understand:

how research could add value to the normal evaluation undertaken, to understand when there is a change in service delivery? (Participants Group 3)

Across groups, evaluative methodologies were discussed, including health economics approaches that could be used to create research outputs. Finally, this definitional dialogue was added to by a declaration by participants that some items as being “not HSR” but rather “public health research” and hence they excluded these items from their prioritisation.

(3) Addressing the vulnerability of the population and the health workforce

Within most groups, the topic of vulnerable populations emerged as a significant concern. The mental health of the whole of population was raised, as well as the impact on vulnerable (or priority) groups already known to experience poorer care and outcomes in the Australia community. At times, the discussion focused less on researchable questions that should be prioritised and more on discussion about significant societal challenges and responses to the pandemic. One participant expressed concern about understanding the impact of COVID-19 on Aboriginal and Torres Strait Islander communities, saying:

What did happen? In what way were Aboriginal and Indigenous communities engaged in that response? And to what extent was it imposed upon them? From emergency safety perspective? And (we need) to connect with Canada, NZ and other nations with large collective indigenous communities who are highly sensitive … it's about ensuring that we recognise the impact of rural and remoteness as a factor in the dialogue. (Participants)

Groups were concerned about the vulnerability of healthcare workers and that research should prioritise how best to communicate with community members about COVID-19 and preventing infection.

(4) A strong desire to learn from rapid responses and changes across the health system

Participants expressed a strong desire to learn from the changes and adaptations that were made across health services during the pandemic. As participants in Group 5 noted:

The best time to prepare for an emergency is when you aren’t having one. We need to be better at planning for shocks that are coming and are unexpected. (There are) a lot of learnings from the response.

Other participants expressed strong appreciation for changes in collaborative public health research:

COVID has seen an unprecedented collaboration which is something that isnt always so evident in the health sector.

The pace with which change was able to occur in the health system was extraordinary. In terms of a policy action, there is a tension between traditional governance structures – which were able to be bypassed. Which of these changes and flexibility that could be carried forward?

Group members placed emphasis on evaluating rapid changes in practice for the health workforce and what could be done better in the future to *“maximise our surge capacity and to actually look at those gaps”*. One participant said:

The health system came together really quickly and looked at scopes, the practice around ICU and changes to clinical models of care. And we really quickly developed this great protocol for the model of care shifts. And then as we started to move into the peak and down the other side of the of the curve … people started moving back and started defaulting back to what were historical clinical practice models. People were … quite prepared to work outside the traditional areas.

#### Session summation

The roundtable included a final session in which the group chairs reported back to all participants. Participants commented on how impressed they were with the priority setting process and quality of NSW Health’s “policy on the run” that had occurred during the pandemic. Group chairpersons drew attention to discussion about the priorities and reaching a balance between preparedness and conducting clinical activities had been supported by new models of care and asked:

How in the future do we create that balance between preparedness and normal activity?

As an overall summation, it was agreed that prioritising research programs rather than numerous small grants would be one productive way of learning from the changes experienced by the health system during COVID19.

##### Subsequent decisions taken to fund projects

The second grant funding was announced, and guidelines released five weeks after the workshop. Overall, there was a lack of concordance between the recommended priorities from the roundtable with the round two funding call, with only three of the eight recommended priorities included. These were:

(1) Health services response: Conduct rapid cycle evaluations of major affected clinical services.
(2) Health services response: How do we make sense of the reduced visits to emergency departments and GPs; and,
(3) Mental health service response: What were the determinants of successful interventions to support the mental health needs of vulnerable populations during the COVID response?

Additional priorities included were identifying effective models of care, public health messaging, diagnostics and prevention and therapeutics. However, the introduction to the funding call stated that NSW Health would “prioritise projects that fulfill the following criteria: research using a system-wide approach so that findings can be scaled in NSW” and “research that has high potential for translation into policy and practice”. Situating the funding call within a research translation context suggests that the roundtable’s emphasis on a strong desire to learn from rapid responses was reflected in the overall ethos of the funding call.

An overwhelming volume of round two funding applications were submitted, anecdotally reported as about 250 submissions. The successful applications to Round Two were across topic areas of mental health impacts (four grants, including vulnerable populations), effective models of care (two grants addressing the needs of rural populations), public health messaging (one about vaccine public health messages), with two focused on diagnostics and one about therapeutics.

## Discussion

This priority setting roundtable demonstrates that, during the COVID-19 pandemic, it was possible to bring together a highly experienced and senior group of healthcare stakeholders to identify research priorities through a mechanism that was collegial and efficient. Recommended priorities for research were formulated through the consensus process that emphasised learning from health service response to the pandemic, addressing mental health and conduct research into the impacts on vulnerable groups in the population. The roundtable participants recognised an immediate opportunity to guide research priorities, whilst operating under pressure and a need to respond quickly.

A purpose of this manuscript was to articulate what lessons were learned about what might be useful and generalisable for future approaches to HSR priority setting. Firstly, we tested a modified Nominal Group Technique methodology in a virtual meeting format in a novel way that minimised the required time investment by senior health services executives, highly experienced clinicians and researchers to run a priority setting roundtable. The process of thorough preparation in reviewing potential items and carefully selecting items for deliberation, as well as preparing resources for the chairpersons to engage participants in discussion were significant contributors to success of the roundtable. It allowed for reviewing and rating priorities whilst engaging in rich discussion about the scope of HSR and evaluation, as well as consideration of what was important to learn from health services responses to the COVID-19 pandemic. Indeed, participants eagerly engaged and wanted to see research funded that would enable health service innovations to be documented and sustained beyond the pandemic. Our experience thus provides a pragmatic contribution to priority setting literature.

A second lesson is that the COVID-19 pandemic has highlighted a need for more rapid and larger scale approaches to health services research to spur their development (Vindrola-Padros *et al.*, 2020; Glasziou *et al.*, 2020) as well as legitimising a strong and overt link between science and public policy. This may well result in long term changes to research approaches (Richardson *et al.*, 2020) and policy-makers’ views of the role and value of research. The authors note that is the intent of HSR is to improve health outcomes by highlighting the needs and demands of patients and communities, with research into the best approaches for supply of effective, equitable and efficient health care (Health Services Research Association of Australia and New Zealand, 2022). Thus, HSR must be amenable to action-oriented evaluative methods, which is consistent with international calls across knowledge synthesis (Neil-Sztramko *et al.*, 2021), implementation science (Glasgow and Chambers, 2012) and Learning Health Care systems (Riley *et al.*, 2013) over the past 2 decades (Zucco *et al.*, 2021).

A third lesson is that the research-policy nexus involves complex, messy and unpredictable processes that do not necessarily align with a linear process of developing research priorities. The authors recognise that research engagement activities such as roundtables are one activity that contributes to decision-making by policymakers alongside expert advice, stakeholder interests, economic conditions, resource constraints, legislative infrastructure and political ideologies (Redman *et al.*, 2015). The limited adoption of the recommendations into the subsequent research call and projects that were funded may have been disappointing for some participants. It is recognised there are risks in “over-doing” priority-setting and co-production exercises as “participating stakeholders need to see allocation of resources to support problem solving for it to be meaningful” (Cooke *et al.*, 2015). Otherwise, priority-setting can create false expectations or hopes. In this work, priority setting may have been more acceptable to stakeholders because of the highly efficient process used to solicit their input.

In terms of lessons learned about approaches to priority setting, the value or return on investment from conducting research priority setting exercises has rarely been assessed. There is still more to be learned about what influence priority setting exercises has on outcomes. For example, when published priority-setting exercises in nutrition were reviewed and their authors surveyed, influence on research seemed limited; these papers were poorly cited and authors felt they lacked impact on funders (Hawwash *et al.*, 2020). Lack of follow-up or implementation of strategies involving funders, researchers and government appears to be common (Yoshida, 2016).

Finally, as would be expected with a group of individuals bringing the different perspectives of health service managers, clinicians and researchers there were disagreements about the roles of health services research and evaluation. A detailed epistemological analysis of the distinction between evaluation and research is beyond the scope of this article. However, the view that evaluation is constituted as being more practical are widely held (Wanzer, 2020). The paucity of competitive funding for health service research in Australia, unlike what has been funded in the UK (Kings Fund and Health Foundation have provided sustained HSR funding over many years) meant that some participants were not able to draw on previous experiences of valuable local health service research – in particular, examination of Australian policymaking and management processes have been rare. Health service research can draw from (and contribute to) theory to make stronger recommendations and we consider HSR is crucial to support the major investment decisions that will establish a value-based resilient health system.

The limitation of this work includes that members of the public were not involved in initial generation of items, nor where they engaged in the roundtable meeting. This exclusion was far from ideal (Hunter *et al.*, 2016) but was a response to the significant time constraints. Further, the authors were not aware of any consumer organisations that had offered training for consumer or community members to address COVID research priorities. The workshop was conducted at a time when very few cases had occurred in the Australia community. Consideration of public values and input into priorities for HSR about the impact of COVID-19 is an area for further research and ethical considerations about this topic have been addressed in the literature (Straiton *et al*., 2020).

## Conclusion

This work reports on a priority setting roundtable exercise to guide decision making by NSW Health and provide recommendations about investments in future health services research (HSR) funding. We have described the successful application of the priority setting methodology. It is possible to efficiently engage a large group using an existing priority setting methodology in an online meeting format. The application of this new online context appeared to be valued by time poor participants who were focused on managing complex health services in a crisis. We have extracted generalisable lessons that could inform strategic priority setting for HSR. There was a lack of concordance between the recommended priorities from the roundtable with only three of the eight recommended priorities included in the round two funding call. We acknowledge that activities such as roundtables are only one of a myriad of factors contributing to decision-making about funding priorities by policymakers. Future investigations of the experiences of health services manager, policymakers, researchers and consumers would be valuable in shaping future research priorities about the impacts of the COVID-19 pandemic.

## Supplementary Material



## Figures and Tables

**Table 1:** Professional groupings of roundtable participants

Professional groups	*N*	%
Ministry of Health Representatives	37	42
Academics	14	16
Clinician-Researchers	13	14
Chief Executives (Pillars, LHD/LHN, PHN)	13	14
Deputy CEO, COO or Director	6	7
Program Managers, Executive Staff	6	7
*Total*	*89*	*100*
*Sex*		
Female	49	55
Male	40	45
*Total*		*100*

**Source(s):** Authors’ original research data/created by the authorship team

**Table 2:** Overview of the priority setting process

Project scope and preparation phase	Pre-roundtable planning	Roundtable reporting	Post-roundtable
*Establishing governance and team members across agencies* - Engaged team and setting of purpose and objectives *Identify a guiding framework and review best practice approaches to priority setting* - COVID-19 System Shock Framework - Two authors reviewed priority setting methods *Initial collection of items for prioritisation* - NSW Health Round 1 (R1) priorities - NSW Health Round 2 (R2) draft priorities identified through internal consultation process - Review of Academy Health report on rapid cycle evaluation *Collating and categorising priorities* - Two team members created an initial list - Deletion of irrelevant items, grouping under domains - Translate topics into statements, refinements made by other team members	*Selection of a framework and a prioritisation methodology* - Modified Nominal Group Technique - Develop and define criteria for prioritising items - Agreement on rating process *Identification of participants* - Engagement across NSW Health, Health Research Translation Centres, Health Services Researchers and clinicians across universities and health sector - Pre-roundtable survey sent out two days prior *Defining clear processes for translation of methodology from traditional to online delivery* - Instructions for group leaders, secretariat - Participant instructions - PowerPoint slides, IT resources	*Engagement on the day* - Chairperson outlined program and roundtable purpose - Priority setting tasks were explained to determine most important items - Breakout groups convened and chaired; secretariat assigned to ensure smooth transitions into groups - Recording of sessions for transcription *“Success” in rating items across groups* - Process of engagement over Zoom, scoring and ranking methodology - Team noted strengths and limitations of online engagement. *Revision of scores and whole of group discussion* - Whole group discussion to reach consensus before closing *Rapid preparation of report* - Final list of priorities submitted to NSW Health	*Factors that impacted on final selection of items* - Not a strategic investment - Not health services research - Undergoing internal evaluation *Final priorities for R2* - Priorities released by NSW Health five weeks after roundtable - Applications close three weeks after release *Lessons learned* - Meaningful engagement across sectors into the priority setting process. Engagement of a large group of stakeholders via Zoom was feasible and achievable within tight time constraints - The COVID-19 pandemic has highlighted the need for more rapid and larger scale approaches to HSR and legitimised a strong and overt link between science and public policy - The research-policy nexus involves complex, messy and unpredictable processes that do not necessarily align with a linear process of formally developing research priorities - There is still more to be learned about what influence priority setting exercises has on outcomes

**Source(s):** Authors’ original research data/created by the authorship team

## Supplementary Material

The supplementary material for this article can be found online.
