# Supplementary material for: COVID-19 research response to immediate demands: setting priorities with key stakeholders to enable health services research in NSW, Australia
Source: J Health Organ Manag. 2024 Sep 17;38(9):344–59. doi: 10.1108/JHOM-03-2023-0059 (PMC11440472; doi:10.1108/JHOM-03-2023-0059)
Supplement: Supplementary file 1 [file jhealthorganmanag-38-0344-s001.docx]

**Supplementary File:**

**Table III: Full list of items rated and ranked during the roundtable.**

| **Specific examples**  *Action-oriented research questions*  **For ratings, 1 = Not Significant, 7 = Very Significant** | **Significance of topic** | | **Round 2 COVID-19 grants** | | **Ranking Round 2 COVID-19 grants**  **(Ranked within groups)** |
| --- | --- | --- | --- | --- | --- |
|  | **Average pre-workshop survey score** | **Breakout Group Score** | **Average pre-workshop survey score** | **Breakout Group Score between 1 and 7** |  |
| **Groups 1A and 1B** |  |  |  |  |  |
| 1. Conduct rapid cycle evaluations of major affected clinical services such as elective surgery, cancer treatments and transplants, management of chronic illness (e.g., pain), palliative care, mental health. (How have they been impacted by COVID-19? Are there changes that should be sustained? Assess health economic impact of reconfigured services. Are there remedial actions we need to take to protect patients who missed out on care? Which services are a priority for such evaluations?) | 6 | 7 | 6 | 7 | **1** |
| 1. Undertake fine-grained modelling of the impacts and costs of localised and time-varying COVID-19 public health measures in the ‘spot fire’ phase of the pandemic (e.g., localised intensive testing, localised lockdowns). | 5 | 7 | 5 | 7 | **2** |
| 1. What are the essential features of successful (fast and efficient) ‘step up’ response to a pandemic? (e.g., Was the creation of individual plans by LHDs and hospitals the best and most efficient use of resources? What does capacity mean in a normal scenario, and how can it be increased in a crisis? How do capacity and possible adaptations vary for urban, rural and remote services?) | 5 | 6 | 5 | 7 | **3** |
| 1. How do we determine when we can ‘step-down’ in health service preparedness for a pandemic? | 5 | 3 | 5 | 4 | Not reported (NR) |
| 1. How do we make sense of the reduced visits to emergency departments and GPs during the COVID-19 scare? (Are there strategies that can help more patients manage outside the hospital system in ‘normal times’? Consider cohorts of patients that frequently present that have reduced their presentation and understand what strategies the patient employed rather than attending ED and determine if the patient experienced a deterioration in their health and wellbeing.) | 6 | NR | 6 | 4 | NR |
| 1. How have changes in models of care for alcohol and other drugs treatment been impacted? a) What are the impacts in terms of access, clinical outcomes and safety? b) What are the impacts on consumer outcomes and experience? c) What are the impacts on vulnerable communities (e.g. pregnant women, those experiencing domestic violence, children under protection services, Aboriginal and Torres Strait Islander people, youth in out-of-home-care, culturally and linguistically diverse communities, homeless people)? | 5 | NR | 5 | 4 | NR |
| 1. Develop models of care to predict and respond to increases in morbidity and mortality in mental health during the recovery phase. | 6 | NR | 6 | 4 | NR |
| **Group 2** |  |  |  |  |  |
| 1. Mental health issues related to quarantine and isolation, and social distancing, particularly in groups that are already at risk of isolation in the community (CALD, disability sector). | 5 | 7 | 5 | **7** | **1** |
| 1. Investigate the efficacy of communications to patients during the public health emergency e.g., their understanding of the safety of service delivery, and changes in health services (e.g., telehealth) and the efficacy of education about COVID-19 including personal safety measures. | 5 | 6 | 5 | **6** | **2** |
| 1. What lessons can be learned from examining community behaviours, including acceptance and compliance with restrictions? Why was Australian public compliance with social distancing and working at home policies impressive? How can we ensure similar responsiveness for second waves or other pandemics? | 5 | 4 | 5 | **5** | **3** |
| 1. Mental health impacts of the COVID-19 response and recovery phases across the lifespan, including impacts in education, employment etc. | 5 | 6 | 5 | 6 (merged with item #8) | (merged with item #8) |
| **Group 3** |  |  |  |  |  |
| 1. Study what was/is needed for a significant increase in use of telehealth (infrastructure and communication skills, challenges of having to educate patients as part of consultations, equity issues). | 6 | 4 | 6 | **7** | **1** |
| 1. Evaluate the impact of the new MBS items to enable telehealth including reimbursement, uptake, acceptability to consumers, appropriateness for different medical conditions. | 6 | 6 | 5 | **6** | **2** |
| 1. What are the effects of virtual care approaches to health care delivery on clinical and patient-reported outcomes, and clinician experience? | 6 | 2 | 6 | **7** | **3** |
| 1. Evaluate the COVID-19 induced scale-up and the future sustainability of hospital in the home for patients with COVID-19 and serious chronic conditions. | 6 | 3 | 6 | 5 | 4 |
| 1. What are the major supply chain risks for health in NSW? How can we work to ameliorate these? | 5 | 5 | 5 | 5 | 4 |
| 1. What is the health and other data we need to share in a health system crisis? How can we facilitate this sharing for future crises and develop a more effective learning health system during ‘normal operations’? | 5 | 1 | 5 | 4 | 6 |
| **Group 4** |  |  |  |  |  |
| 1. Track how equity of care and access were implemented broadly in response to COVID-19. How were the needs of priority populations within wider state-wide COVID-19 planning addressed? Document the effectiveness of strategies that engaged vulnerable groups and at-risk populations in public health messages and understanding appropriate access to services. 2. E. Evaluate the Aboriginal COVID-19 Management Plan, including the impact on cultural safety and effectiveness of care. | 5 | 6 | 5 | 6 | **1** |
| 1. **Workforce:** Evaluate the extended scope of practice that has occurred internationally in response to COVID-19 and to a lesser extent in Australia and consider how this could be supported/facilitated to provide surge capacity for future crises. 2. **C**. Measuring and reducing the mental health impact of COVID-19 in health care workers and other essential workers. | 5 | 5 | 5 | 5 | **2** |
| 1. **Group 4** A. Examine impact of COVID-19 on undergraduate health students and specialist training and future service delivery implications of this. | 5 | 5 | 4 | 6 | **3** |
| 1. D. How socio-economic determinants of health change post-COVID-19? | 5 | 3 | 5 | NR | NR |
| 1. G. What are the successful innovations and mistakes that emerged as a result of geographic variation we need to learn from? (How can we best involve rural communities in pandemic planning?) | 5 | NR | 5 | NR | NR |
| 1. H. How can we best weigh infection control concerns against the financial and environmental consequences of extreme usage of PPE? | 5 | NR | 5 | NR | NR |
| **Groups 5A and 5B** |  |  |  |  |  |
| 1. How did changes to NSW Health and Department of Health policy and practice enable rapid responses to support health service change? Understand how the strategic thinking, collaboration, and work practices of the agencies that came come together to achieve the outcomes (and the enablers and barriers). | 5 | 6 | 5 | 6 | **1** |
| 1. **NEW ITEM:** Evaluate the direct and indirect costs and funding models for telehealth, teleconsultations, and virtual models of care. | 5 | 6 | 5 | 6 | **2** |
| 1. Have best practice research governance processes been undertaken during COVID-19? How can we ensure these are both strong and agile for future health system shocks or the next pandemic? How have human ethics research committees coped with the demands for changes to existing approvals, influx of new studies (related to COVID-19)? | 5 | 6 | 4 | 2 | **3** |
| 1. What policy redesign will better support rapid responses to future health system shocks or pandemics? | 4 | 5 | 4 | 6 | 4 |
| 1. What has been the large-scale impact of the direct and indirect costs of COVID-19 to the NSW health system and NSW economy. | 5 | 5 | 4 | 6 | 5 |
| 1. **NEW ITEM:** How do we design and implement systems to ensure equitable distribution of treatments/services during a pandemic? (e.g., logistics, supply chain issues, etc) | 4 | 5 | 4 | 4 | 6 |
| 1. How can primary care, the community health sector and residential aged care facilities be integrated more expediently into future emergency health system responses? | 5 | 4 | 5 | 3 | 7 |

Source(s): Authors’ original research data /created by the authorship team

**Table IV: Selection, refinement and prioritisation of items across the project**

|  | **Stages of development** | | | |
| --- | --- | --- | --- | --- |
|  | **Project scope and preparation phase** | **Pre-roundtable planning** | **Roundtable reporting** | **Post-roundtable** |
| **Data sources and brief description** | | | | |
|  | **Initial collection of items for prioritisation**  NSW Health Round 1 (R1) priorities: 4 domains containing 18 items.  Domain headings:   - Prevention of infection - Diagnostics research - Treatment, including antivirals and immunosuppressive agents - Public and population health.   Specific exclusions were listed, such as epidemiological studies and modelling, vaccine research and development. The first three domains contained limited health services research topics.  NSW Health Round 2 (R2) draft priorities [identified through internal consultation process]: 5 domains containing 17 items.  Domain headings:   - Identifying effective models of care - Mental health impact of COVID-19 - Public health messaging - Prevention and therapeutics - Diagnostics | **A pre-roundtable survey** consisting of 6 themes and 33 items based on Academy Health items (DeCosta et al., 2020) was distributed to participants:   - Health system response to pandemic, including reconfiguration of clinical services (7 items) - Patient and Community Experience including behavioural insights and communications (4 items) - Medical technologies and information systems (6 items) - Health workforce, including needs and training (3 items) - Health system values (5 items) - Health policy, governance and whole of government response and impacts (8 items) | Eight items (summarised below)   1. **Health system response:** Was the “cascaded planning approach” the best way to plan a response to the pandemic? 2. **Health services response:** Conduct rapid cycle evaluations of major affected clinical services. 3. **Health services response:** How do we make sense of the reduced visits to emergency departments and GPs during COVID-19? 4. **Mental health service response:** What were the determinants of successful interventions to support the mental health needs of vulnerable populations during the COVID response? 5. **Medical technologies:** Which digital modality should we be using for which purpose/cohort? 6. **Health services response - priority populations**: How were the needs of priority populations within wider state-wide COVID-19 planning addressed? 7. **Workforce:** Evaluate the extended scope of practice developed in response to COVID-19 8. **Primary Care:** How can primary care, the community health sector and residential aged care facilities, non-health government agencies and NGOs be integrated more expediently | Final priorities contained in R2 call for funding: 5 Domain headings (17 items)   - Identifying effective models of care (3 items) - Mental health impact of COVID-19 (4 items) - Public health messaging (3 items) - Prevention and therapeutics (1 item) - Diagnostics (6 items)   **General principles for R2 grants:**  For all research topics, NSW Health will prioritise projects that fulfil the following criteria:   - Research using a system-wide approach so that findings can be scaled in NSW. - Research that has high potential for translation into policy and practice. - Studies measuring clinically important outcomes. - Large, multidisciplinary and/or collaborative projects and trials. - Research that includes consideration of health equity between different population groups. |

Source(s): Authors’ original research data /created by the authorship team; Items selected from the Academy Health report (DeCosta et al. 2020).
